# Longevity and skeletal muscle mass: the role of IGF signalling, the sirtuins, dietary restriction and protein intake

**DOI:** 10.1111/acel.12342

**Published:** 2015-04-10

**Authors:** Adam P Sharples, David C Hughes, Colleen S Deane, Amarjit Saini, Colin Selman, Claire E Stewart

**Affiliations:** 1 Stem Cells, Ageing & Molecular Physiology Unit, Research Institute for Sport and Exercise Sciences (RISES), Exercise Metabolism and Adaptation Research Group (EMARG), Liverpool John Moores UniversityTom Reilly Building, Liverpool, L3 3AF, UK; 2 Department of Neurobiology, Physiology and Behavior, University of CaliforniaDavis California, CA, 95616, USA; 3 MRC/ARUK Centre of Excellence for Musculoskeletal Ageing Research, School of Medicine, University of Nottingham, Royal Derby HospitalDerby, DE22 3DT, UK; 4 School of Health and Social Care, Bournemouth UniversityBournemouth, BH12 5BB, UK; 5 Department of Physiology and Pharmacology, Karolinska InstitutetStockholm, 171 77, Sweden; 6 Glasgow Ageing Research Network (GARNER), Institute of Biodiversity, Animal Health and Comparative Medicine, College of Medicine, Veterinary and Life Sciences, University of GlasgowGlasgow, G12 8QQ, UK

**Keywords:** AKT, AMPK, cachexia, calorie restriction, FOXO, high-protein diets, IGF-I, IRS-1, lifespan, longevity, MAFBx, mTOR, MURF, regeneration, sarcopenia, satellite cells, SIRT, SkM, TSC

## Abstract

Advancing age is associated with a progressive loss of skeletal muscle (SkM) mass and function. Given the worldwide aging demographics, this is a major contributor to morbidity, escalating socio-economic costs and ultimately mortality. Previously, it has been established that a decrease in regenerative capacity in addition to SkM loss with age coincides with suppression of insulin/insulin-like growth factor signalling pathways. However, genetic or pharmacological modulations of these highly conserved pathways have been observed to significantly enhance life and healthspan in various species, including mammals. This therefore provides a controversial paradigm in which reduced regenerative capacity of skeletal muscle tissue with age potentially promotes longevity of the organism. This paradox will be assessed and considered in the light of the following: (i) the genetic knockout, overexpression and pharmacological models that induce lifespan extension (e.g. IRS-1/s6K KO, mTOR inhibition) versus the important role of these signalling pathways in SkM growth and adaptation; (ii) the role of the sirtuins (SIRTs) in longevity versus their emerging role in SkM regeneration and survival under catabolic stress; (iii) the role of dietary restriction and its impact on longevity versus skeletal muscle mass regulation; (iv) the crosstalk between cellular energy metabolism (AMPK/TSC2/SIRT1) and survival (FOXO) versus growth and repair of SkM (e.g. AMPK vs. mTOR); and (v) the impact of protein feeding in combination with dietary restriction will be discussed as a potential intervention to maintain SkM mass while increasing longevity and enabling healthy aging.

## Sarcopenia: demographics and impact on quality of life in humans

Life expectancy is increasing rapidly in many countries. As a consequence, there are a greater proportion of older people making up our global population. In the UK, 10 million people are currently over 65 years of age, with the latest projections suggesting that this will increase to 19 million people by 2050 (Cracknell, 2013). Age is the primary risk factor for a multitude of pathological conditions, including Alzheimer’s disease, cardiovascular disease, type II diabetes and sarcopenia. Sarcopenia is the age-related loss of Skeletal Muscle (SkM) mass and function (Rosenberg, 1997). Muscle loss is evident in sedentary humans at 25 years of age, with a 10% loss in peak lean SkM mass at 40 years of age, which increases to 40% at 70 years of age (Porter *et al*., 1995). Indeed, from age 50, muscle mass is lost at a rate of 1–2% per year (Hughes *et al*., 2001). This loss impacts negatively on functional and metabolic performance, maximal strength and muscle quality (Renault *et al*., 2002; Morse *et al*., 2005a,b; Rossi *et al*., 2008). Importantly, loss of functional capacity in skeletal muscle with age is strongly correlated with decreased quality of life and increased frailty, morbidity and early mortality (Rantanen *et al*., 2003). Given that approximately 40–50% of the population over 80 years of age suffers from sarcopenia, this condition has been recognized as a major geriatric clinical disorder (Cruz-Jentoft *et al*., 2010). Thus, ameliorating age-related SkM wasting is of high clinical importance if we are to improve quality of life and ultimately reduce the socio-economic impact of sarcopenia.

### Overview and Rationale

This review will focus on the cellular and molecular mechanisms that underpin age-related muscle loss and will debate the trade-off that may occur between skeletal muscle maintenance and survival into old age versus whole organism life/healthspan. This concept emerges from the body of research investigating the molecular modulators of aging. It focuses on genetic knockout (KO) of IRS-1 and p70S6K1 as well as transgenic models such as FOXO, SIRT1 and finally pharmacological modulation including mTOR inhibition and sirtuin activation. All of these models have been shown to extend both lifespan and healthspan. Importantly however, all of these pathways are also inextricably shared with those that modulate skeletal muscle mass maintenance. Therefore, this review will seek to discuss the hypertrophic, degradative and sirtuin pathways in relation to their modulatory regulation of lifespan, healthspan and muscle cell survival particularly in inflamed aged environments. Finally, the potential importance of optimizing dietary restriction and amino acid uptake to ameliorate the reduction in SkM mass while promoting healthy aging will be discussed.

## Insulin-like growth factors (IGFs) and skeletal muscle

### Overview of IGF’s and their role in skeletal muscle mass regulation

The insulin-like growth factor (IGF) family consists of the ligands, IGF-I and IGF-II, the type I and type II IGF cell surface receptors, six specific high-affinity binding proteins (IGFBP-1 to IGFBP-6), IGFBP proteases and other IGFBP-interacting molecules (Holly *et al*., 2000). They have a wide range of biological functions including embryonic, foetal and adult SkM development (reviewed in Stewart & Rotwein, 1996a). *In vivo* rodent studies have shown that KO of IGF-I, IGF-II or the IGF-I receptor (IGF-IR) results in animals that are phenotypically small for their gestational age with significant decreases in SkM mass and neonatal lethality (Nabeshima *et al*., 1993; Lau *et al*., 1994; Stewart & Rotwein, 1996a,b). Alternatively, increasing circulating IGF-I expression in transgenic mice results in SkM hypertrophy (Matthews *et al*., 1988). Furthermore, KO of IGF-IIR also results in SkM overgrowth; as IIR acts as a clearance receptor for IGF-II, thus its removal leads to an increase in circulating IGF-II and subsequent hypertrophy (Lau *et al*., 1994). Our group has extensively characterized the multifaceted roles of the IGF system where they are fundamental in the proliferation, survival, differentiation and hypertrophy of primary human and mouse SkM cells (Stewart *et al*., 1993; James *et al*., 1996; Stewart *et al*., 1996; Stewart & Rotwein, 1996b; Stewart *et al*., 1999a,b; Foulstone *et al*., 2001, 2003a,b, 2004; Grohmann *et al*., 2005; Saini *et al*., 2008; Stewart & Pell, 2010; Al-Shanti & Stewart, 2011; Saini *et al*., 2012; Sharples *et al*., 2013; Player *et al*., 2014) (Reviewed in Scime & Rudnicki, 2006). Skeletal muscle-derived IGF-I is also important in adult muscle hypertrophy, as demonstrated using liver IGF-I-deficient (LID) mice (Matheny *et al*., 2009). In this study, despite an 80% reduction in total circulating levels of IGF-I in LID versus control (L/L) mice, following 16 weeks of hypertrophy inducing resistance exercise there was no difference in locally produced IGF-I mRNA or IGF-IR activation between groups (Matheny *et al*., 2009). Despite these compelling data, the importance of IGF-I in mechanical load-induced hypertrophy following resistance exercise and the development of animal models of nonphysiological hypertrophy have been recently debated. This controversy is reviewed by our group elsewhere, and it not the focus of this current review (Stewart & Pell, 2010; Sharples & Stewart, 2011).

### Reductions in IGF-I and associated signalling in aging skeletal muscle

With sarcopenia, a 33% reduction in circulating IGF-I (Benbassat *et al*., 1997) and a 45% decline in SkM-derived IGF-I mRNA are observed in older (70 ± 0.3 years) vs. younger (20 ± 0.3 years) human males (Leger *et al*., 2008). A corresponding attenuation in downstream intracellular signalling targets involved in protein synthesis with age has also been described. These include reductions in the activity of PI3K, Akt, mTOR, p70S6K1, 4E-BP1 and EIF2B in older vs. younger counterparts (Terada *et al*., 1994; Welsh *et al*., 1997; Pallafacchina *et al*., 2002; Cuthbertson *et al*., 2005; Leger *et al*., 2008). With impairments of these signalling pathways also observed with age following muscle contraction (Fry *et al*., 2011), a recent study using mouse models attempted to recapitulate declining human serum IGF-I concentrations with age. It should be noted that in rodents, serum IGF-I levels are consistently high and do not decrease until very old age when sarcopenia is observed, whereas in humans, serum IGF-I is highest during adolescence and declines earlier in the life course, starting in middle age and paralleling the onset of sarcopenia. This study suggested that mice with reduced serum IGF-I at 1 year of age had significantly deteriorated healthspans. They exhibited increased liver weight and inflammation and increased incidence of hepatic tumours. Importantly, in SkM tissue, increased oxidation of proteins was observed, indicative of increased oxidative stress (Gong *et al*., 2014), overall suggesting an important role for IGF-I in reducing some, but not all (see below), age-associated pathologies.

We have recently developed and begun to characterize the roles of the IGFs, their receptors and modulatory binding proteins in an *in vitro* murine cell model of SkM aging via the following: (i) comparisons of parental (older) vs. daughter (younger) cell populations and (ii) multiple population doublings as a way of artificially aging cells (Sharples *et al*., 2010, 2011, 2012, 2013). These studies demonstrated that IGF binding protein levels are increased in cells that display aging phenotypes via mechanisms that ultimately reduce the activity of Akt (Sharples *et al*., 2011, 2013). These observations correspond with impaired differentiation and hypertrophy of myotubes (Sharples *et al*., 2010, 2011, 2012; Deane *et al*., 2013). These phenotypes are also observed in primary human SkM cells isolated from aged vs. young donors (Collins *et al*., 2007; Bigot *et al*., 2008; Pietrangelo *et al*., 2009; Beccafico *et al*., 2010). These effects correspond with a loss of myogenicity (Hidestrand *et al*., 2008) in the face of unchanged telomere length and telomerase activity (O’Connor *et al*., 2009). Together, the majority of evidence (both *in vitro* and *in vivo*) therefore points towards the need for IGF-I and activation of its downstream signalling pathways to maintain skeletal muscle mass across the lifespan.

## Reduced Insulin/Insulin-like-Growth Factor Signalling (IIS): enhanced longevity vs. reduced muscle mass in aging skeletal muscle

### IGF and Insulin Receptor Substrate (IRS-1)

Reductions in IGF-I activity with age are associated with reductions in SkM size and function. However, reduced signalling through the IIS pathway is also associated with increased lifespan and healthspan in model organisms (Clancy *et al*., 2001; Holzenberger *et al*., 2002; Barbieri *et al*., 2003; Tatar *et al*., 2003; Giannakou & Partridge, 2007; Piper *et al*., 2008; Selman *et al*., 2008; Vallejo *et al*., 2009; Kenyon, 2011; Selman *et al*., 2011). For example, both female and male mice globally lacking insulin receptor substrate 1 (*Irs1*^*−*^*/*^*−*^) are long lived (Selman *et al*., 2008, 2011). Female mice lived 32% longer compared to wild-type controls, equating to a mean lifespan of 971 days in the *Irs1*^*−*^/^*−*^ mice compared with 738 days in wild-type control animals. Interestingly, *Irs1*^*−*^/^*−*^ mice showed resistance to several parameters associated with aging, including bone, skin, metabolic, immune and motor dysfunction (Selman *et al*., 2008). Thus, *Irs1*^*−*^/^*−*^ mice, in common with several other long-lived models, enjoy a greater period of their life free from various age-associated pathologies (Selman and Withers 2011). Importantly, *Irs-1*^*−*^/^*−*^ mice display reduced growth compared to wild-type animals perhaps due to the important role for IRS-1 in embryonic and postnatal growth (Withers *et al*., 1998, 1999). Furthermore, mice with growth hormone (GH)/IGF-I defects, while phenotypically growth retarded compared with wild-type littermates, also exhibit enhanced longevity, lower DNA mutation frequencies, higher DNA excision repair and secondary attenuation of IIS (Bates & Holder, 1988; Pell & Bates, 1992; Bartke & Brown-Borg, 2004; Bartke, 2005; Garcia *et al*., 2008; Garinis *et al*., 2009; Masternak *et al*., 2009; Page *et al*., 2009).

While there are clear benefits of reduced IIS signalling for lifespan and aspects of healthspan, as eluded to above, reductions in SkM mass correspond with decreases in IGF-I with age. Indeed, some studies suggest that bone, cardiac muscle and other tissues display aged characteristics when IGF-I is impaired (Adamo and Farrar, 2006; Anversa, 2005; Ceda *et al*., 2005; Geusens and Boonen, 2002). Indeed, *Irs1*^*−*^/^*−*^ mice have reduced body weight and fat mass compared to age-matched controls (Pete *et al*., 1999; Selman *et al*., 2008) with reduced gastrocnemius SkM weight that is proportionately greater than the decrease seen in total body weight (Pete *et al*., 1999). *Irs1*^*−*^/^*−*^ mice are, however, more resilient to age-associated osteoporosis compared to controls, which may account somewhat for this discrepancy. A recent study using an inducible liver-derived IGF KO mouse, allowing temporal reductions of IGF of 70% in the serum, showed that lower IGF from the age of 1 year resulted in greater oxidative stress in SkM, accelerated bone loss and reduced lifespan (Gong *et al*., 2014). Indeed, across 31 genetically diverse inbred mouse strains, lower serum IGF-I was associated with enhanced longevity (Yuan *et al*., 2009). Furthermore, human population studies suggest that reductions in IGF-I at young age but elevations at old age might maximize healthy lifespan, reviewed in Yang *et al*. (2005). To the authors’ knowledge, the only study to investigate potential mechanisms of SkM adaptation with IRS-1 loss suggested that it did not affect glucose uptake or GLUT1/4 function in electrically stimulated mouse muscle (Dumke *et al*., 2001). Skeletal muscle mass or synthetic/degradative signalling was, however, not investigated in this study. Overall, it is clear that reductions in IIS enhance lifespan and delay some aging-associated parameters yet perhaps results in small body size that is characterized by both reduced fat mass and potentially, proportionally smaller SkM mass. However, more investigation into SkM mass and the corresponding cellular signalling in *Irs1*^*−*^/^*−*^ mice into old age is required in the near future to understand the potential crosstalk between the mechanisms that control increased lifespan and healthspan while contributing to reductions in SkM mass with age.

### Mammalian target of Rapamycin (mTOR)

In addition to reduced IIS, reduced signalling through the target of rapamycin (TOR) signalling pathway has also been shown to modulate lifespan and increase healthspan in model organisms (Kapahi *et al*., 2004; Kaeberlein *et al*., 2005; Powers *et al*., 2006; Hansen *et al*., 2007; Harrison *et al*., 2009; Anisimov *et al*., 2010; Bjedov *et al*., 2010; Miller *et al*., 2011; Robida-Stubbs *et al*., 2012; Zhang *et al*., 2014). Longevity in humans is also associated with reduced mTOR signalling (Slagboom *et al*., 2011; Passtoors *et al*., 2013). The TOR or mTOR (mammalian target of rapamycin) is, however, a key regulator of SkM growth where it also plays a central role in the crosstalk between growth and metabolism in a wide variety of cell types (Inoki *et al*., 2003) and SKM (most recently see Hamilton *et al*., 2014). Mammalian target of rapamycin regulates its hypertrophic effects in SkM through the phosphorylation of downstream effectors such as P70S6 kinase 1 (S6K1) and eIF4E-binding protein1 (4E-BP1) (reviewed in Schiaffino *et al*., 2013). Their roles in SkM growth following contraction and mechanical load-induced hypertrophy, synergistic ablation, myotube hypertrophy and amino acid sensing are also well defined (Fujita *et al*., 2007; Drummond *et al*., 2009; Willett *et al*., 2009; Goodman *et al*., 2011; Miyazaki *et al*., 2011; Philp *et al*., 2011; Jacobs *et al*., 2013; Hamilton *et al*., 2014). In older people, mTOR becomes less responsive to contraction-induced activation (via resistance exercise), compared with young adults (Fry *et al*., 2011). The activity of mTOR in response to amino acid feeding is also impaired in older individuals, a phenomenon known as ‘anabolic’ resistance (Cuthbertson *et al*., 2005).

Rapamycin-induced inhibition of mTOR has, however, been shown to increase lifespan in yeast, *drosophila* and mice (Powers *et al*., 2006; Harrison *et al*., 2009; Anisimov *et al*., 2010; Bjedov *et al*., 2010; Miller *et al*., 2011; Robida-Stubbs *et al*., 2012; Wilkinson *et al*., 2012). Further, rapamycin diminishes a range of aged-related pathologies (reviewed by Johnson *et al*., 2013b), and despite a contentious study claiming that it does not (Neff *et al*., 2013), the wide consensus is that appropriate modulation of mTOR signalling could be an important route of intervention to slow aging and increase healthspan (reviewed by Johnson *et al*., 2013a). However, in skeletal muscle rapamycin-induced inhibition of mTOR has been shown to impair myogenic differentiation (Willett *et al*., 2009), blunt the anabolic response to overload and nutrients (Goodman *et al*., 2011), with muscle-specific inactivation of mTOR leading to myopathy (Risson *et al*., 2009). These data therefore suggest, perhaps paradoxically, that despite inhibition of mTOR increasing lifespan and improving many age-related pathologies, mTOR signalling plays a critical role in maintaining SkM mass and anabolism. Despite this, the only study that has so far investigated muscle size and function in mice with advancing age, suggests that muscle cross-sectional area and grip/paw strength were unaffected by a 16-month treatment of rapamycin vs. aged-matched controls (Neff *et al*., 2013).

**Fig 1:**
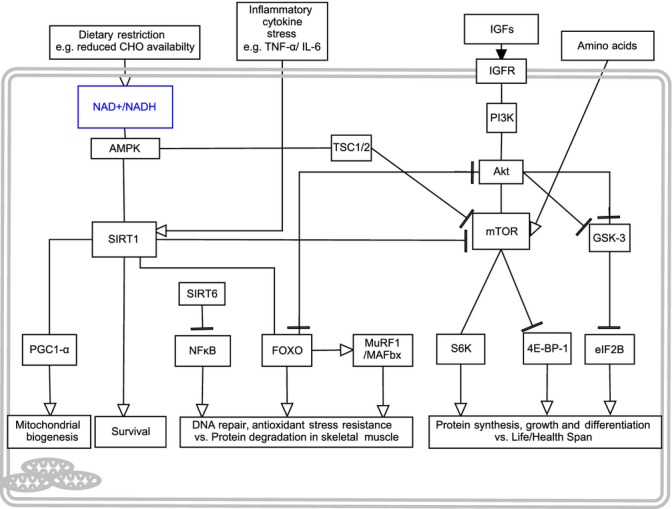
Depicts the extracellular and intracellular signaling molecules involved in the cross-talk between skeletal muscle mass regulation and life/health-span modulation. Genetic or pharmacological suppression of IIS, TOR and Sirtuin pathways increase organism life/health-spans. However, these pathways are fundamental in protein synthesis, growth, differentiation and survival in skeletal muscle into old age. This figure therefore provides the potential molecular and cross-talk modulators for this paradigm of lifespan versus muscle mass maintenance with age.

Similar to rapamycin-induced mTOR inhibition, global deletion of the ribosomal protein S6K1 in mice, a downstream effector of mTOR, also increases lifespan and improves healthspan in mice (Selman *et al*., 2009). In contrast to rapamycin treatment having no impact on muscle size (Neff *et al*., 2013), S6K1(−/−) myotubes are smaller, despite having a normal number of nuclei, and their response to a hypertrophic stimuli of IGF-I or nutrients is blunted (Ohanna *et al*., 2005). Further, deletion of S6K1 in mice induces SkM atrophy even in the presence of high nutrient availability via AMPK activation, where AMPK inhibition in S6K1-deficient myotubes restores SkM growth via increases in myotube diameter and sensitivity to nutrient signals (Aguilar *et al*., 2007). In aged human SkM, S6K1 is downregulated in response to amino acid feeding (Cuthbertson *et al*., 2005) and attenuated in old vs. young rodents during recovery from immobilization-induced atrophy (Morris *et al*., 2004). S6K1 is also reduced in contracting aged SkM in comparison with young muscle, suggesting it plays an important role in SkM protein synthesis, which is hampered with age (Parkington *et al*., 2004; Kumar *et al*., 2009). However, surprisingly little is currently known about whether basal muscle maintenance and function is altered in the context of aging in long-lived mTOR mutant or, as discussed, rapamycin-treated mice. Studies examining protein synthesis, protein degradation and SkM function in long-lived mouse models are urgently required if we are to increase our understanding of the potential trade-off between longevity and muscle function. Depicted in Figure 1 (Fig.1).

## Sirtuins: divergent roles in the modulation of lifespan vs. skeletal muscle mass

### Sirtuins and their roles in aging and longevity

Significant recent research effort has focused on elucidating the various roles of sirtuins (silent information regulator 1–7; Sir1-7) in aging. Sirtuins are a group of seven highly conserved protein deacetylases involved in the process of chromatin remodelling and gene regulation (see Morris, 2013). They have also been shown to have pathophysiological relevance in cancer, obesity, SkM, inflammation and neurodegeneration (Rodriguez & Fraga, 2010; Schug & Li, 2011; Park *et al*., 2012; Donmez & Outeiro, 2013). There is emerging evidence that these proteins may regulate SkM mass, potentially through alterations in IGF-I and associated signalling (discussed below). The metazoan Sir2 proteins are recognized, somewhat controversially, for their role in regulating lifespan in yeast, worms and fruit flies (Kaeberlein *et al*., 1999; Burnett *et al*., 2011; Viswanathan & Guarente, 2011). The rodent homologue of Sir2, SIRT1, does not increase lifespan in mice, although overexpression does improve healthspan (Herranz *et al*., 2010). More specifically, neural-specific SIRT1 overexpression has been shown to increase lifespan and delay aspects of aging relative to wild-type littermates (Satoh *et al*., 2013). Downregulation of SIRT1 also induces an aging phenotype (Sommer *et al*., 2006). Activation, rather than overexpression of SIRT1 using a small molecular activator (resveratrol), reportedly reduces age-related ill health in *ad libitum* fed old mice, if administered from the middle age, it is, however, without impact on lifespan (Pearson *et al*., 2008; Miller *et al*., 2011). Under more pathological conditions, resveratrol administration does extend lifespan, specifically in mice placed on high fat diets (Baur *et al*., 2006). It is worth stating here that resveratrol has pleiotropic cellular targets and therefore, effects cannot always be directly linked to SIRT activation per se and results should be interpreted with this caveat in mind. Interestingly however, SIRT6, when overexpressed in male mice, has also been attributed to increased lifespan (Kanfi *et al*., 2012b) and short-lived phentoypes are evident in SIRT6 KO animals (Mostoslavsky *et al*., 2006).

### Sirtuins and their impact on IGF signalling and skeletal muscle

In terms of SkM growth and protein synthesis, evidence exists, implicating SIRT1 and SIRT6 as negative regulators of IGF-I and downstream Akt/mTOR signalling (Ghosh *et al*., 2010). For example, in mouse neural cells, SIRT1 silencing and overexpression increased and decreased IGF-I and associated Akt signalling, respectively (Sansone *et al*., 2013). Similarly, SIRT6 overexpression in mice has been associated with a reduction in circulating IGF-I (Kanfi *et al*., 2012a). An exciting recent link between SIRT1 and IGF-I has been established in a range of nonskeletal muscle human cell types. When stimulated with exogenous IGF-I for prolonged periods, cells exhibited reduced SIRT1 deacetylase activity, increased p53 acetylation and increased senescence, when compared with cells exposed to acute administration of IGF-I exhibiting increased proliferation (Tran *et al*., 2014). Although speculative, reductions in IGF-I with age could be an attempt to alleviate senescence and maintain SIRT1 activity (Tran *et al*., 2014). In SkM, our group has shown that the induction of apoptosis, by low-dose tumour necrosis factor-alpha (TNF-α) with the addition of IGF-I, is elevated compared with TNF-α administration alone. Death was associated with increased SIRT1 mRNA levels, which when suppressed using SIRT1 siRNA, culminated in exacerbated, not reduced, apoptosis (Saini *et al*., 2008, 2012). Overall suggesting that under conditions of both anabolic and catabolic conflicts, SIRT1 was important to the maintenance of survival in skeletal muscle cells. Therefore, SIRT1 appeared fundamental in negatively regulating IGF-I basally, yet in the presence of inflammatory catabolic stress (Saini et al., 2008, 2012), or where IGF-I exposure was prolonged enough to induce cell death (Tran *et al*., 2014), SIRT1 was important in maintaining survival. It is also worth noting that SRT2104, a synthetic small molecular activator of SIRT1, reduced circulating TNF-α in mice (Mercken *et al*., 2014). Suggesting a potential regulatory loop between SIRT1 and TNF-α, yet this link in SkM is yet to be directly established. This concept is particularly relevant in aging muscle where chronic low-level TNF-α exposure and changing IGF-I concentrations are strongly associated with muscle wasting *in vivo* and the pathologies of sarcopenia and cachexia (Li & Reid, 2000; Meadows *et al*., 2000; Foulstone *et al*., 2001; Greiwe *et al*., 2001; Bruunsgaard *et al*., 2003a,b; Bruunsgaard & Pedersen, 2003; Stewart *et al*., 2004; Grohmann *et al*., 2005; Li *et al*., 2005; Saini *et al*., 2006, 2008, 2010, 2012).

In addition to its role in regulating IGF-I and survival in the presence of aberrant IGF-I, SIRT1 may also play a role in negatively regulating mTOR. SIRT1 (−/−) mouse embryonic fibroblasts (MEFs) and human HELA cells depleted of SIRT1 using shRNAi resulted in elevated mTOR signalling, which was not abolished by leucine deprivation (Ghosh *et al*., 2010). In the same study, SIRT1 activators and inhibitors (resveratrol/nicotinamide) reduced and increased mTOR activity, respectively (Ghosh *et al*., 2010). SIRT1 activation following resveratrol administration in myoblasts inhibited IGF-I-associated signalling (Akt) and abolished leucine-stimulated increases in mTOR (Liu *et al*., 2010). These studies suggest that any changes in SIRT1 with age in response to catabolic stress or nutrient restriction could potentially impact on mTOR function and result in altered regeneration. Overall, these data present potential negative regulation by SIRT1 on pathways such as Akt/mTOR linked to SkM growth. On the contrary, recent work by Hong *et al*. (2014) suggested that SIRT1 and SIRT2 deacetylate the substrate of mTOR, S6K, specifically on mTOR-dependant phosphorylation site Thr-389. In this case, acetylation blocked S6K activation and thus, deacetlyation by the sirtuins may actually be involved in the phosphorylation of S6K (Hong *et al*., 2014). Furthermore, in cardiac muscle, SIRT1 can also deacetylate Akt and PDK, enabling binding to phosphatidylinositol 3,4,5-trisphosphate [PIP(3)], and thus its localization to the membrane where PDK can subsequently facilitate Akt phosphorylation (Sundaresan *et al*., 2011). Sirtuin activation, however, specifically in SkM tissue or cells through overexpression in rodent models or supplementation of resveratrol/its analogues in humans, requires further investigation to decipher its role in negatively or positively regulating SkM mass. Importantly, based on evidence described above, the reductions in IGF-I seen with age could be an attempt to increase SIRT1 to harness its role in cell survival especially when under a catabolic cytokine stress (e.g. TNF-α) that as mentioned above, is chronically elevated in the circulation and skeletal muscle with age (and discussed in more detail directly below).

### Sirtuins and their role in survival and differentiation under catabolic stress in skeletal muscle cells

Despite this apparent trade-off with survival vs. growth, our group has shown that activation of SIRT1 in murine myoblasts following resveratrol administration can begin to rescue differentiation of SkM cells following catabolic stimulation by TNF-α (Saini *et al*., 2012). This is important when considering that TNF-α is chronically increased in the aging circulation and that it is produced by muscle itself (Greiwe *et al*., 2001; Bruunsgaard *et al*., 2003a,b; Bruunsgaard & Pedersen, 2003). In agreement with our group, *res*veratrol can reverse the negative impact of TNF-α on myotube hypertrophy (Wang *et al*., 2014). Similarly, activation of SIRT1 using SRT2104 attenuated SkM mass losses of the gastrocnemius and soleus in mice following 2 weeks of hindlimb unloading (Mercken *et al*., 2014). SRT2104 also extended lifespan, without reducing SkM weight into old age (Mercken *et al*., 2014). Therefore, as well as an important role in myoblast survival, SIRT1 may also be involved in maintaining adequate differentiation, hypertrophy and attenuating atrophy *in vivo* during stress stimuli such as those experienced with chronic inflammation or disuse.

Finally, it is important to consider that changes in the [NAD^+^]/[NADH] ratio occur during skeletal muscle differentiation and this changing ration in turn can regulate SIRT1 (Sartorelli & Caretti, 2005). A reduction in the [NAD^+^]/[NADH] ratio coincides with skeletal myogenesis, whereas an increase is associated with impaired myogenesis (Fulco *et al*., 2003). It is clear, however, that differences prevail in terms of derived data. Indeed, Fulco *et al*. (2008) suggested that increasing SIRT1 activity in mouse and human SkM cells impaired differentiation and myosin heavy chain production (Fulco *et al*., 2003, 2008), which differs from our work with TNF-α, but complements more recent unpublished work where under control conditions, resveratrol increased proliferation in both control and artificially aged myoblasts but impaired differentiation (Deane CS, Hughes DC, Sharples AP, unpublished). An increase in proliferation, inhibition of p21cip and p27kip and a reduction in differentiation following SIRT1 overexpression in rat myoblasts have also been previously reported (Rathbone *et al*., 2009). Therefore, despite its proposed negative regulation of IGF-I/Akt/mTOR, SIRT1 seems to be fundamental to SkM cell survival, enabling proliferation and impairing differentiation under control conditions, yet protecting differentiation under conditions of stress. Importantly, the impact of activating SIRT in aged SkM cells/tissue basally or under stress remains to be fully determined especially, we hypothesise, in situations of dietary restriction that directly regulate the NAD/NADH ratio and impact on SIRT expression.

### Sirtuins: regulators of longevity and survival vs. activators of protein degradation in SkM via FOXO transcription factors

In addition to its role in SkM proliferation, SIRT1 has also been implicated in controlling protein degradative pathways, specifically via forkhead box protein O (FoxO) transcription factors. These transcription factors are involved in targeting and activating members of the ubiquitin proteasome, such as muscle atrophy F-box (MAFbx/atrogin1), muscle RING finger 2 (MuRF1), and autophagy–lysosome pathways involved in protein degradation (Sandri *et al*., 2004; Edstrom *et al*., 2006; Sandri, 2008). SIRTs have been shown to activate both FOXO3a gene expression and deacetylate FOXO3a, thereby activating FOXO DNA binding and elevating the expression of target genes such as p27(Kip1), manganese superoxide dismutase and Bim, proteins associated with cell cycle arrest, oxidative stress and apoptosis respectively (Brunet *et al*., 2004; Wang *et al*., 2007; Jacobs *et al*., 2008). The activation of FOXO transcription factors by the SIRT family appears to, however, impair the ability of FOXO to promote cell apoptosis, instead shifting its function towards oxidative stress resistance and DNA repair (Brunet *et al*., 2004; Greer & Brunet, 2005; Wang *et al*., 2007). It is also well established that overexpression of FOXO can extend lifespan in *drosophila* (Giannakou *et al*., 2004; Min *et al*., 2008; Alic *et al*., 2014). Interestingly, in TNF-α-stimulated SkM cells the activation of SIRT1 via resveratrol restored Akt/mTOR/S6K and 4E-BP1 signalling and reduced FOXO1 but not FOXO3a protein levels, all of which were unchanged basally (Wang *et al*., 2014). Therefore, the role for SIRT1 activation on FOXO3a in SkM tissue with age requires further investigation. FOXO1 and its role in oxidative stress resistance in aging SkM also requires attention, especially following catabolic stress or dietary restriction where SIRT1 elevation is associated with survival. It is worth mentioning here that class I histone deactylases (HDACs) (sirtuins are class III HDACS) have also been linked with activating FOXO3a and the SkM-atrophy programme (via MAFbx/atrogin-1) during nutrient deprivation and disuse-induced atrophy (Beharry *et al*., 2014). Potentially this suggests that FOXO1 and FOXO3a are modulated by class I and class III HDACs, respectively, and this may account for some of the discrepancy detailed above. The role of the SIRTs in SkM is intriguing and warrants further investigation, specifically the promotion of longevity via resistance to oxidative stress vs. increased protein degradation with aging.

### Sirtuins and NF-_K_β and their roles in longevity and skeletal muscle loss with age

While discussing protein degradation above, it is worth noting that SIRT6 has been associated with modulating lifespan via nuclear factor κB (NF-_K_β) signalling (Yeung *et al*., 2004; Kanfi *et al*., 2012b). The inhibition of NF-_K_β delays DNA damage, cellular senescence and oxidative stress during aging (Tilstra *et al*., 2012). However, in SkM, NF-_K_β is another important protein where cytokine and oxidative stress signalling converge to reduce myoblast differentiation, induce atrophy and increase protein degradation (Langen *et al*., 2001; Hunter & Kandarian, 2004; Lu *et al*., 2012). SIRT6 attenuates NF-_K_β signalling through histone deacetylation of NF-_K_β gene promoter regions and suppresses those genes associated with senescence and aging (Kawahara *et al*., 2009). The deletion of SIRT6 in KO mice also results in shortened lifespan and significantly reduced body weight, suggesting an important developmental and postnatal role for this protein:protein interaction (Mostoslavsky *et al*., 2006). Studies by our laboratory suggest that inhibition of NF-_K_β can promote delayed myoblast apoptosis in the presence of TNF-α (Stewart *et al*., 2004). It is, however, worth noting that there was no change in NF-_K_β during disuse atrophy (2 weeks hindlimb suspension) even in the presence of SRT2104 (Mercken *et al*., 2014). Interestingly, very recent work suggests SIRT activation in murine models via SRT2104 causes a reduction in the ratio of phosphorylated NF-_K_β to total protein (Mercken *et al*., 2014). This therefore suggests that SIRT1 and SIRT6 may be important in reducing NF-_K_β. Overall, SIRT1 and/or SIRT6 may regulate lifespan as a consequence of reduced IGF-I signalling and potentially attenuate the effects of inflammatory NF-_K_β signalling.

## Effect of Dietary Restriction (DR) on longevity and skeletal muscle mass

Calorie restriction is defined as a reduction in energy intake, while maintaining nutrient intake, relative to that consumed normally by individuals with free (*ad libitum*) access to food (Selman, 2014). For the purposes of this review, dietary restriction (DR) will incorporate both calorie restriction and those interventions in which macro/micronutrients are altered without any overall change in energy intake. DR is the most reproducible intervention, to date, to extend medium and maximum lifespan in various model species (Mair & Dillin, 2008; Speakman & Selman, 2011; Selman, 2014). In mice, there seems to be a strain-specific association with DR and longevity, and in primates, the link between lifespan extension and DR may also be confounded by genetic heterogeneity (reviewed by Selman, 2014). Nevertheless, DR reduces incidence and severity of various pathological conditions in rodents and primates, which are leaner, and display reductions in insulin resistance, glucose intolerance, cognitive decline and immune dysfunction (Masoro *et al*., 1982; Barger *et al*., 2003; Selman *et al*., 2005; Mattison *et al*., 2012), indicating DR *per se* is beneficial for health.

### Trade-off between cellular energy metabolism and growth in skeletal muscle with dietary restriction

The intuitive impact of chronic DR on SkM mass is that over time, absolute muscle mass decreases. This is not surprising if you consider that in the presence of nutrient restriction, the cell shifts away from growth in an attempt to survive. Further, protein from SkM can provide energy during severe nutrient restriction. One of the first studies to demonstrate this and to establish the molecular link between AMPK energy sensing and cellular growth through mTOR/S6K signalling was that of Inoki and collegues (Inoki *et al*., 2003). Using various cell types (HEK293, MEF, EEF, LEFs) under starvation conditions, they reported increased AMPK activity and phosphorylated tuberous sclerosis 2 (TSC2). The TSC2 inhibited mTOR and other substrates, including S6K, 4EBP-1 and EIF2, which resulted in reduced cell size and growth rates. The role of TSC2 in this process was confirmed in TSC2 KO cells, which grew and maintained normal size in the presence of starvation. The AMPK activation of TSC2 and inhibition of mTOR therefore appears central in responses to energy deprivation. Fascinating but perhaps unintuitively, given the data thus far, DR appears to delays or prevent age-related loss of SkM mass in rats and rhesus monkeys via attenuation of DNA damage, proteosomal machinery, autophagy, inflammatory signalling and mitochondrial abnormalities (Aspnes *et al*., 1997; Phillips & Leeuwenburgh, 2005; Hepple *et al*., 2008; McKiernan *et al*., 2011). Indeed, short-term DR can potentially increase SkM stem cell availability and subsequent SkM repair following cryo-injury in young and old mice (Cerletti *et al*., 2012). In a recent *in vivo* study, chronic DR (by 30% of recommended daily intake) for a period ranging from 4 to 20 years (mean 9.6 years), resulted in reduced IGF-I levels, and a threefold reduction in Akt mRNA/ 30–50% reduction in Akt activity, together with increased FOXO3a and FOXO4 expression (Mercken *et al*., 2013). These changes in FOXO were reported to modify several genes linked to longevity including genes associated with stress resistance, antioxidants, DNA repair, protein turnover and cell death (Mercken *et al*., 2013). In SkM however, this shift away from growth towards stress resistance, would potentially reduce protein synthesis and increase degradation over time (Sandri *et al*., 2004; Edstrom *et al*., 2006). Furthermore, superoxide dismutase 2 (SOD2) expression, a transcriptional target of FOXOs, was increased under DR, as was DNA damage-binding protein 1 (DDB1), both key regulators of DNA repair. Further, cyclin D2 was significantly downregulated during moderate DR, as a fundamental orchestrator of cell cycle progression for proliferation or growth (Mercken *et al*., 2013). Interestingly, DR in rats also reduced levels of the inflammatory cytokine TNF-α and associated signalling (Phillips & Leeuwenburgh, 2005). These studies therefore suggest that chronic moderate (∼30%) DR results in transcriptional reprogramming, which shift cellular regulation from growth to maintenance/repair and lifespan activities, while potentially reducing local inflammation. Perhaps most importantly, humans and mice on DR diets had higher lean SkM mass-to-fat mass ratios (Mercken *et al*., 2013). Therefore, there is potentially an optimal level of DR which has the beneficial effect of longevity, while perhaps preventing growth but not inducing muscle loss. Although overall SkM mass is likely to be reduced by long-term DR, the ratio of lean mass to fat mass may be greater and total body weight maybe reduced, a signature conducive of reduced metabolic disease risk. It remains to be determined, however, whether chronic DR changes SkM strength or the proportions of extracellular matrix to muscle tissue, or alters contractile properties and force per cross-sectional area/muscle quality. Indeed, the influence on force production following DR could be affected by fibre type, as type I fibres were ∼62% larger after DR (30% DR for 12 years) in rhesus monkeys vs. control. Furthermore, in this study it was observed that there was delay in type II fibre atrophy with age (McKiernan *et al*., 2011). So while data of long-term studies are limited, they do suggest potential for both longevity and muscle health.

Despite this body of work, several other studies oppose these findings. For example, although different to sustained DR, Lee and Goldberg investigated the impact of acute fasting in mice and showed that this resulted in a reduction in SIRT1 activity and an increase in the atrogenes MuRF-1 and atrogin-1, which ultimately led to a significant decrease in SkM mass (Lee & Goldberg, 2013). Dietary restriction (−30%) for 6 weeks, in combination with exercise, also reduced gastrocnemius SkM weight and cross-sectional area in comparison with similarly exercised mice under *ad libitum* feeding (Park *et al*., 2013). However, it should be noted that this study did not include a DR or *ad libitum* alone group. This does, however, highlight the temporal role of short-duration fasting vs. longer duration DR and the modulation of SIRT1 (McKiernan *et al*., 2012; Mercken *et al*., 2013). DR in combination with physical activity and its impact on SkM phenotypes therefore requires further investigation. Finally, it is unlikely that DR is a pragmatic intervention for humans, given that there is a considerable level of motivation and restraint required, where DR mimetics maybe more practical as reviewed previously by Selman *et al*. (Selman, 2014).

## Roles of amino acid feeding or high-protein diets in association with calorie restriction: potential impact on skeletal muscle mass vs. disease and longevity

One of the issues with DR is the contribution of total calories from carbohydrates vs. proteins. Most studies do not differentiate between the two. It is well established that protein intake can enhance muscle protein synthesis in a dose-responsive manner in young and old adults (Cuthbertson *et al*., 2005; Moore *et al*., 2009). Furthermore, increasing dietary protein can help maintain SkM mass during periods of disuse (reviewed in Wall & van Loon, 2013) and induce greater increases in skeletal muscle hypertrophy following chronic supplementation when combined with exercise (resistance) vs. exercise alone (meta-analysis Cermak *et al*., 2012). Indeed, there is substantial support to suggest that with DR, overall weight loss is no different with higher protein intakes vs. DR alone (Sacks *et al*., 2009; de Souza *et al*., 2012). With some acute trials showing that fat mass decreases while SkM is spared (Krieger *et al*., 2006), importantly, exercise in combination with higher protein content in DR diets seems to have a SkM maintaining effect (Garthe *et al*., 2011; Josse *et al*., 2011; Mojtahedi *et al*., 2011), without negative impact on markers of mitochondrial biogenesis, albeit after acute fasting in humans (Taylor *et al*., 2013). Interestingly, undertaking DR that is protein rich reduces both body mass and percentage body fat, with associated reductions in circulating insulin and IGF-I levels (Maestu *et al*., 2010), alluding to potential benefits for lifespan while potentially maintaining SkM mass. Supplementation with branched-chain amino acids (BCAAs) such as leucine, isoluecine, valine or metabolites of leucine such as β-hydroxy-β-methylbutyrate (HMB) have become a favoured intervention as they have been shown to activate mTOR and protein synthesis in SkM to a greater extent compared with other essential/nonessential amino acids (Atherton *et al*., 2010; Pimentel *et al*., 2011; Churchward-Venne *et al*., 2012; Salles *et al*., 2013). Leucine alone can activate protein synthesis in humans to the same extent as whey protein and mixed essential amino acids plus leucine when administered 1–3 h postresistance exercise (Churchward-Venne *et al*., 2012). However, the requirement for whey protein for optimal protein synthesis 3–5 h postexercise is acknowledged (Churchward-Venne *et al*., 2012; Phillips, 2014). Previously, Mourier and colleagues observed that DR in human males (wrestlers) when combined with supplementation of mixed BCAAs led to a reduction in total body mass and fat mass (−17.3%), although SkM mass was unchanged (Mourier *et al*., 1997). This suggests a potential role for BCAAs in maintaining SkM mass under DR conditions. Furthermore, a recent study highlighted that HMB attenuated the loss of SkM mass observed following DR in murine exercise models (Park *et al*., 2013). Mice underwent exercise at 6 m.min^−1^ run for 1 h, three times a week alone or combined with HMB and/or DR. The HMB animals had higher lean mass than the training alone group. Grip strength decreased under DR, but was maintained in DR mice supplemented with HMB. Interestingly, gastrocnemius mass and myofibre cross-sectional area were greater with HMB in the presence of a DR diet compared to DR alone, albeit there were no data reported for either ad *libitum* or HMB alone supplemented mice (Park *et al*., 2013). This latter finding was also associated with the reduced ubquitin ligase, MAFbx, alluding to reduced protein degradation. Surprisingly however, Akt and mTOR mRNA were elevated under DR conditions in SkM. Speculation based on evidence presented in above sections suggests this may be due to increased SIRT1, yet this hypothesis requires further investigation. Therefore, in the light of the above discussion it would be prudent to investigate, on a background of DR, how AMPK and SIRT1 (energy sensing) change in the presence of BCAAs and the way in which they impact on Akt/mTOR (growth) via the molecular modulators of TSC1/TSC2 (discussed above and seen in Fig.1).

Finally, it is important to note that increased protein intake, especially BCAAs, stimulates targets such as mTOR and S6K, which are downstream of IIS, the precise signalling which is reportedly suppressed to enable longevity and to reduce age-related disease. This therefore contributes to the recently debated paradigm whereby downstream IIS signalling is still activated, yet independently of IGF binding to its receptor, and thus protein synthesis in SkM mass may be maintained with increased protein intake during aging. However, it has been conversely suggested that increased protein intake may increase incidence of diseases, such as cancer, and thus impact negatively on longevity (Renehan *et al*., 2004). Indeed, it is known that cancer patients who do not respond to chemotherapy or are end-stage patients have reduced protein diets that, while potentially adding to the chronically inflamed milieu that causes SkM loss (cachexia), can slow tumour progression. Examples include animal models where DR can attenuate tumorigenesis via inhibition of mTOR, whereas leucine feeding can increase pancreatic tumour growth in both lean and overweight mice (Vellai *et al*., 2003; Bjornsti & Houghton, 2004; Hursting *et al*., 2010; Lashinger *et al*., 2011; Liu *et al*., 2014). Restricting the amino acid methionine can also limit tumour growth, and both methionine and essential amino acid restriction increase lifespan in rodents (Richie *et al*., 1994; Miller *et al*., 2005; Emran *et al*., 2014; Sinha *et al*., 2014). Overall, these studies suggest caution for cancer patients and amino acid supplementation, even those who suffer with muscle loss (Liu *et al*., 2014). The role of higher protein diets with age and the impact on disease risk and early mortality have recently received a high level of attention. Cohorts of 6381 adults aged 50 and over were studied for their habitual dietary intake and macronutrient composition with corresponding disease and mortality incidence (Levine *et al*., 2014). Between the ages of 60 and 65, those who reported high animal-derived protein intake had a 75% increased risk in overall mortality and a fourfold increase in cancer risk during the subsequent 18 years. If aged over 65 years of age, however, higher protein intake was associated with reduced cancer risk, but a fivefold increased risk of diabetes. These results therefore suggested that a low-protein diet is potentially beneficial in midlife; however, the benefits reduce with age. In an attempt to compliment these studies with mechanisms, high-protein diets were implemented in middle-aged mice, where the increase in GH/IGF signalling observed was associated with increased progression of tumours. The authors did, however, suggest that low protein impacted negatively on SkM mass in aged mice (Levine *et al*., 2014). In agreement with this study, an investigation published in the same issue as that by Levine *et al*. using a Geometric Framework approach to investigate the contributions of protein-to-carbohydrate ratios and their association with increased longevity in mice, suggested that healthy aging is not as a consequence of high-protein low calorie diets, but low-protein (especially BCAAs) diets, with the remaining macronutrients being made up of carbohydrate rather than fat (Solon-Biet *et al*., 2014). Also, data by Levine *et al*. have been scrutinized in terms of the methodological design. For example, 24-h dietary recalls suggesting up to 18 years of habitual diet are potentially not appropriate to account for lifelong habitual dietary intake. Furthermore, the grouping of the low- to high-protein categories [based on Institute of Medicines’ (IOM) Acceptable Macronutrient Distribution Range] has also received attention, where the low-protein group would probably be classed as protein deficient. It is also worth stating that in the total cohort (50 years and over), the level of protein intake was not associated with differences in all-cause, cancer or CVD mortality. Importantly, however the study did find a significant association between the subjects aged 50–55, higher protein consumption and cancer/mortality. Amongst 2253 subjects, the risk of cancer and mortality was increased in the high-protein subjects who also had higher IGF-I serum levels. It is indeed, established that people in the highest circulating IGF-I quintiles are at the highest risk of developing cancer (Hankinson *et al*., 1998; Kaaks *et al*., 2000; Giovannucci *et al*., 2003) and the role of IGF-I and associated signalling in cancer cells and tumour development is fairly robust (Pollak *et al*., 2004; Guevara-Aguirre *et al*., 2011). It is important to note that these are similar pathways to growth/amino acid stimuli required for SkM maintenance with age. The future paradigm we should be addressing would therefore be the trade-off between maintenance of SkM mass vs. longevity, potentially at the expense of age-related diseases.

## Conclusion

The understanding of aging and the development of interventions to increase healthy lifespan have been greatly aided by the development of genetic mutants for IIS, TOR and sirtuin pathways as well as the use of pharmacological agents known to act on these pathways. However, all of these pathways are fundamental in regulating the trade-off between survival and maintenance vs. growth, particularly in skeletal muscle where age-associated losses in SkM mass and function are observed with advancing age. This provides a paradigm in which there is potentially reduced regenerative capacity within SkM tissue with age in an attempt to promote longevity of the organism and survival within the tissue. Optimizing dietary restriction (DR) or using DR mimetics in combination with amino acid administration may be critical interventions to help attenuate SkM loss with advancing age, while enabling healthy aging.

## Author contributions

Sharples, AP is the corresponding author who instigated/conceptualized the review and wrote the first draft of the manuscript, amended all drafts and worked extensively on the final draft as well as in creating the final figure to complete the manuscript. Hughes, DC and Deane, CS wrote sections of the review with Sharples, AP and significantly inputted to the writing and reading of the manuscript. Saini A, Selman C and Stewart, CE contributed extensively to editing the manuscript while contributing both very valuable comments and important insights and additions throughout.

## Funding

No funding information provided.

## Conflict of interest

None declared.
